# Drug Delivery System Based on pH-Sensitive Biocompatible Poly(2-vinyl pyridine)-b-poly(ethylene oxide) Nanomicelles Loaded with Curcumin and 5-Fluorouracil

**DOI:** 10.3390/polym12071450

**Published:** 2020-06-28

**Authors:** Camelia-Elena Iurciuc-Tincu, Monica Stamate Cretan, Violeta Purcar, Marcel Popa, Oana Maria Daraba, Leonard Ionut Atanase, Lacramioara Ochiuz

**Affiliations:** 1 Department of Pharmaceutical Technology, Faculty of Pharmacy, “Grigore T. Popa” University of Medicine and Pharmacy, University street, no. 16, 700115 Iaşi, Romania; camelia_tincu83@yahoo.com (C.-E.I.-T.); cretanmonica@yahoo.com (M.S.C.); ochiuzd@yahoo.com (L.O.); 2 Department of Natural and Synthetic Polymers, Faculty of Chemical Engineering and Protection of the Environment, “Gheorghe Asachi” Technical University, 700050 Iaşi, Romania; marpopa2001@yahoo.fr; 3 National R&D Institute for Chemistry and Petrochemistry—ICECHIM, Splaiul Independentei 202, 6th district, 060021 Bucharest, Romania; purcarvioleta@gmail.com; 4 Academy of Romanian Scientists, Splaiul Independentei Street No. 54, 050085 Bucharest, Romania; 5 Faculty of Dental Medicine, “Apollonia” University of Iasi, Pacurari street, no. 11, 700355 Iași, Romania; maria.mary2019@yahoo.com

**Keywords:** pH-sensitive micelles, poly(2-vinyl pyridine)-b-poly(ethylene oxide), curcumin, 5-fluorouracil, hemocompatible, biocompatible

## Abstract

Smart polymeric micelles (PMs) are of practical interest as nanocarriers for the encapsulation and controlled release of hydrophobic drugs. Two hydrophobic drugs, naturally-based curcumin (Cur) and synthetic 5-fluorouracil (5-FU), were loaded into the PMs formed by a well-defined pH-sensitive poly(2-vinyl pyridine)-b-poly(ethylene oxide) (P2VP_90_-b-PEO_398_) block copolymer. The influence of the drug loading on the micellar sizes was investigated by dynamic light scattering (DLS) and it appears that the size of the PMs increases from around 60 to 100 nm when Cur is loaded. On the contrary, the loading of the 5-FU has a smaller effect on the micellar sizes. This difference can be attributed to higher molar mass of Cur with respect to 5-FU but also to higher loading efficiency of Cur, 6.4%, compared to that of 5-FU, 5.8%. In vitro drug release was studied at pH 2, 6.8, and 7.4, and it was observed that the pH controls the release of both drugs. At pH 2, where the P2VP sequences from the “frozen-in” micellar core are protonated, the drug release efficiencies exceed 90%. Moreover, it was demonstrated, by in vitro assays, that these PMs are hemocompatible and biocompatible. Furthermore, the PMs protect the Cur against the photo-degradation, whereas the non-ionic PEO corona limits the adsorption of bovine serum albumin (BSA) protein on the surface. This study demonstrates that these pH-sensitive PMs are suitable for practical utilization as human-safe and smart, injectable drug delivery systems.

## 1. Introduction

The emergence of nanotechnology has had a profound impact on clinical therapeutics in the last two decades. Compared to conventional chemotherapeutic agents, nanoscale drug carriers have demonstrated the potential to address some of these challenges by improving treatment efficacy while avoiding toxicity in normal cells, due to features such as a high selective accumulation in tumors via the enhanced permeability and retention (EPR) effect and active cellular uptake. These materials include polymeric micelles (PMs), polymer-DNA complexes (“polyplexes”), liposomes, and other nanostructured materials for biomedical applications, which are collectively called nanomedicines [1,2,3,4].

PMs have been used as promising nanocarriers for drug delivery due to their favorable properties, such as excellent biocompatibility, prolonged circulation time, favorable particle sizes (10–100 nm), and their EPR effect. PMs are obtained by the self-assembly of individual polymer chains (unimers) of amphiphilic block or graft copolymers when they are directly dissolved in an aqueous or non-aqueous solution (dissolution method) above a critical micelle concentration (C.M.C) or a critical micelle temperature (C.M.T) [2,5,6,7]. In addition to this basic method, other micellization techniques are described in the literature: film hydration, precipitation, and dialysis methods starting from a common solvent. Among these methods, the dialysis technique is the recommended, as it avoids the formation of large micellar aggregates [5,6]. These micellar structures have proven their ability to include and to deliver poorly water-soluble drugs, improve drug stability, and have good penetration and site-specificity, leading to an enhanced therapeutic efficacy [8]. Furthermore, micelles can be economically produced on a large scale, which is an important practical advantage [9].

The most common hydrophilic block used to form the micellar shell is the U.S. Food and Drug Administration-approved poly(ethylene oxide) (PEO). Moreover, different types of hydrophobic blocks have been sufficiently studied as drug loading cores. Examples include: poly(L-amino acids) and biodegradable poly(esters), which include poly(glycolic acid), poly(D lactic acid), poly(D,L-lactic acid), copolymers of lactide/glycolide, and poly(ε-caprolactone) [5,6]. The choice of hydrophobic block is largely dictated by its compatibility with the drug, when the drug is physically loaded, and by the kinetic stability of the micelles. The physical incorporation or solubilization of drugs within PMs is generally preferred over micelle-forming polymer-drug conjugates, especially for hydrophobic drug molecules. For a given drug, the extent of incorporation is a function of factors that also control the micelle size and/or aggregation number. Such factors include the ratio of hydrophobic to hydrophilic block length and the copolymer molecular weight [10]. Further enhancement of the therapeutic efficacy of these micellar systems can be achieved by exploring the advantage of stimuli-responsive drug delivery systems [11,12].

Nowadays, naturally-based therapeutic active principles are attracting intense industrial attention owing to their insignificant cytotoxicity on normal cells, reduced side-effects, and costly price compared to their synthetic counterparts [13]. Among these products, curcumin (Cur), the major active component of turmeric (*Curcuma longa* L.), is intensively used in an usual manner as a spice and as a coloring agent in yellow mustards, cosmetics, pharmaceuticals, and hair dyes. Recently, Cur, which is a naturally occurring polyphenolic phytoconstituent, has attracted the interest of the pharmaceutical industry due to its anticancer, antioxidant, anti-inflammatory, hyperlipidemic, antibacterial, wound healing, and hepatoprotective properties [14]. Until now, pre-clinical data have demonstrated that Cur induces apoptosis of cancer cells in different tissues or organs, such as colon, breast, prostate, and lungs [15]. Despite all these promising therapeutic benefits, its clinical efficacy is limited due to poor oral bioavailability, which has been attributed to the very low aqueous solubility and extensive first-pass metabolism [16].

5-Fluorouracil (5-FU), a commonly administered chemotherapeutic drug, exhibits increased bioavailability and a versatile generation of antineoplastic properties being used as a potent drug against solid tumors. Moreover, 5-FU blocks the methylation reaction of deoxyuridylic acid to thymidylic acid, and its use is restricted by its low aqueous solubility, systemic toxicities, severe gastrointestinal toxicities, hematologic side effects, and severe bone marrow disturbances [17,18].

In order to improve the therapeutic efficiency and to reduce the side effects of these highly hydrophobic compounds, Cur and 5-FU were loaded in various PMs which are readily soluble in aqueous media and have an improved dosing, especially in the case of intravenous administration [19,20,21,22]. However, as far as one can tell from the literature, no studies exist concerning the loading of these drugs in pH-sensitive nanomicellar systems based on poly(2-vinyl pyridine)-b-poly(ethylene oxide) (P2VP-b-PEO) block copolymers. In addition to thermo-responsive block copolymers micelles in the aqueous medium, mainly those based on poly(*N-*isopropyl acrylamide) (PNIPAM), pH-sensitive micelles have attracted significant interest [5,6,23]. Among these copolymers, the micellization of a series of poly(2-vinyl pyridine)-based di- block and tri-block copolymers and their complexation with anionic surfactants as a function of pH were recently studied [24,25]. For a series of three pH-sensitive P2VP-b-PEO copolymers, it has been demonstrated that stable nanomicelles, with sizes ranging from 35 to 150 nm as a function of the molar mass of the copolymer, can be obtained at pH > 5 with a water-insoluble “frozen-in” P2VP core and a PEO corona, whereas at lower pH, which leads to the protonation of the P2VP sequences, double hydrophilic block copolymer unimers are formed. This is a direct proof that the pH may control the release of a drug-loaded in the micellar core. Moreover, in another study it was demonstrated that P2VP sequences form interpolymer complexes in organic solvents by hydrogen bonds [26]. This feature of the P2VP sequence may increase the drug loading efficiency. Furthermore, it can be mentioned that the P2VP-based copolymers were used as efficient macromolecular stabilizers for the preparation of stable non-aqueous emulsions which can be diluted with water at pH 1 and/or 7 [27,28].

The general aim of this study was to obtain Cur and 5-FU-loaded P2VP_90_-*b*-PEO_398_ nanomicelles with high loading efficiency and controlled release. A second objective was to validate these drug-loaded micellar systems as human-safe injectable drug delivery systems by carrying out a series of investigations concerning their interactions with the blood components and the in vitro cytotoxicity on human fibroblasts, and also to evaluate the in vitro protein adsorption on the micellar surface. Finally, the antioxidant activity of the Cur-loaded in the micelles was compared with that of free-Cur in order to evaluate the protective effect of PMs against the photo-degradation under UV irradiation.

## 2. Experimental

### 2.1. Materials

Curcumin powder (extracted from *Curcuma Longa*) (Mn = 366.38 g/mol), 5-fluorouracil (Mn = 130, 077 g/mol), Tween 20, bovine serum albumin (BSA), dimethylsulfoxide (DMSO), and methanol were purchased from Sigma Aldrich (Steinheim, Germany). All the solvents were used as received and without further purification. Human dermal fibroblasts cell line (HDFa) and the necessary supplies (antibiotic cocktail: penicillin and streptomycin, non-essential amino acids, trypsin solution, and fetal bovine serum—FBS) for in vitro cytotoxicity assay were purchased from Thermo Fisher Scientific (Waltham, MA, USA).

The molecular characteristics of the P2VP_90_-b-PEO_394_ block copolymer (Mn,_total_ = 27,000 g/mol and Đ = 1.09) prepared by sequential anionic polymerization and used in this study were provided elsewhere [24]. However, it must be noted that the values given as subscripts represent the polymerization degrees of each block. This copolymer sample was chosen among the three copolymers studied in the previous study [24] as it has an intermediary total molar mass.

### 2.2. Preparation of Free and Drug-Loaded P2VP-b-PEO Nanomicellar Systems

The preparation of nanomicellar systems was carried out by a dialysis method. In a typical procedure, 250 mg of P2VP_90_-b-PEO_394_ block copolymer was added in 50 mL of dimethylsulfoxide (DMSO) solution and stirred at room temperature. After the complete dissolution of the copolymer, the solution was directly dialyzed, for 24 h, against 1 L of ultrapure water using cellulose dialysis membranes (molecular weight cut off: 12 kDa, manufacturer, Sigma Aldrich, Steinheim, Germany). The water was changed five times during the dialysis process. After this period, the micellar solution from the dialysis bag was collected, frozen, and then lyophilized in order to obtain a dry powder. This powder was stored at −4° before further use.

The same procedure was used for the preparation of Cur and 5-FU-loaded micellar systems with the difference that at the solution of the block copolymer in DMSO was added 25 mg of Cur or 5-FU, respectively. The micellar sizes were determined by dynamic light scattering (DLS) measurements which were carried out on a Malvern Nano-ZS Zetasizer (Worcestershire, UK) equipped with a 4 mW He–Ne laser operating at a wavelength of 532 nm and a scattering angle of 173°. The software package of the instrument calculates, by using the Stokes–Einstein equation, the Z-average diameter, which is an intensity weighted size average and the polydispersity index (PDI) of the sample. In order to determine the mean diameter of the particles, the data were collected in automatic mode and 5 consecutive measurements were performed for each experiment.

### 2.3. Fourier Transform Infrared Spectroscopy Analysis

FTIR vibrational spectra of lyophilized Cur and 5-FU-loaded PMs, free PMs, Cur, and 5-FU were recorded using an attenuated total reflectance (ATR) device with a Bruker VERTEX 70 spectrometer (Vertex 70, Bruker, Billerica, MA, USA) in the scanning range of wavelength 400–4000 cm^−1^. Numbers of scans and resolution were fixed to 50 and 4 cm^−1^ respectively. The peak assignments are provided in Tables S1 and S2. For lyophilization, liquid suspensions in double distilled water (20 mL) were frozen with liquid nitrogen and freeze died using a Labconco freeze drier (2.5 L, Kansas City, MO, USA) for 24 h.

### 2.4. Drug Encapsulation Efficiency

In order to determine the encapsulation efficiency of both Cur and 5-FU, two calibration curves were constructed in DMSO, using different concentrations of drugs, and their absorbance values were recorded on a nanodrop spectrophotometer at the wavelength of 435 nm for Cur and 267 nm for 5-FU.

A known amount of the drug-loaded micelles, as powder, was dissolved in 3 mL of DMSO, and then these solutions were transferred to dialysis membranes. These membranes were immersed in 15 mL DMSO, in Erlenmeyer flasks, and kept under stirring in a water bath at 37 °C in the dark for 48 h in order to extract the active ingredients. The amount of both Cur and 5-FU from the micelles was spectrophotometrically quantified, based on their calibration curve, using a UV spectrometer. Drug encapsulation efficiency (DEE) and drug loading efficiency (DLE) were calculated using Equations (1) and (2), respectively:

(1) $$DEE\;\left(\%\right)=\frac{Amount\;of\;drug\;in\;micelles}{Amount\;of\;added\;drug\;}\times 100$$

(2) $$DLE\;\left(\%\right)=\frac{Amount\;of\;drug\;in\;micelles}{Amount\;of\;added\;polymer\;and\;drug\;}\times 100$$

Three determinations were performed for each sample and the errors were ± 0.3%.

### 2.5. In Vitro Drug Release Kinetics

*In vitro* drug release kinetics were studied in phosphate buffer solution (PBS) at three different pH values: at pH = 7.4, which is specific for blood and colonic fluids; at pH = 6.8, simulating the intestinal fluid; and at pH = 2 (solution prepared from 10 mM NaCl and 0.1 N HCl) which simulates the stomach pH.

A given amount of lyophilized drug-loaded micellar powder (10 mg) was dispersed in 5 mL PBS, at a given pH value, and then added to a dialysis membrane. The samples thus prepared were immersed in 20 mL PBS, having the corresponding pH value, under stirring, at 37 °C, in the dark in order to avoid the photo-degradation of the Cur. At definite time intervals, samples were extracted in order to quantify spectrophotometrically the amount of the released Cur, at a wavelength of 425 nm, and the amount of 5-FU at 266 nm. As Cur and 5-FU are hydrophobic substances, with low solubility in aqueous media, 1% (*w*/*w*) Tween 20 was added to the release medium. The release kinetics were studied until an equilibrium was reached.

To study the release mechanism of both Cur and 5-FU from the PMs, the Ritger–Peppas kinetic model (Equation (3)) was considered to fit the experimental data [29].

(3) $$\frac{M_{t}}{M_{\infty }}=k\times t^{n}$$

where, *M_t_*/*M*_∞_ is the percentage of drug release at time *t*; *k* is the kinetic constant; and *n* is the diffusion exponent. This equation was applied only for *M_t_*/*M*_∞_ < 60%.

### 2.6. Antioxidant Activity of Curcumin

The method concerning the investigation of the antioxidant activity of Cur was adapted from the one described by Choi et al. [30].

In a typical experiment, a Cur stock solution was prepared by dissolving 25 mg curcumin in 50 mL methanol. Starting from this stock solution, several dilutions were performed in order to obtain the final Cur solutions with the following concentration values: 2, 4, 6, 8, 10, 14 µg/mL. In the test tubes, 3 mL of each solution at a given concentration was added. Then, to the Cur solutions from the tubes was added 1 mL of 0.1 mM DPPH solution. The samples thus prepared were vortexed for 20–30 s and the absorbance was read after 30–40 min using a UV spectrophotometer at a wavelength of 515 nm. Ascorbic acid was used as a standard. The absorbance values were converted to the percentage of antioxidant activity (the percentage of DPPH free radicals inhibition) using Equation (4):

(4) $$I\left(\%\right)=100-\left[\frac{A_{s}-A_{b}}{A_{c}}\times 100\right]$$

where A_s_ is the absorbance value of the sample, A_b_ is the absorbance value of the blank solution prepared from 1 mL of methanol and 3 mL of Cur solutions of different concentrations (the absorbance was measured for each concentration), and A_c_ is the absorbance value of the control solution prepared using 3 mL of DPPH solution and 1 mL of methanol. The calibration of the spectrophotometer was performed with methanol.

In order to determine the antioxidant activity of Cur loaded into PMs, non-irradiated and UV irradiated samples at 365 nm for 30 min were used. The samples were irradiated in order to demonstrate the capacity of the PMs to protect the Cur against the photo-degradation. In order to extract the active principles, the micelles were disintegrated by the addition of 5 mL of methanol. This solution was put in dialysis membranes and kept under stirring in a water bath at 37 °C for 24 h in closed containers (Erlenmeyer flasks) in the dark. The amount of Cur extracted from the micelles was determined spectrophotometrically based on the previously drawn calibration curve. IC50, which is used in order to quantify the antioxidant activity, represents the sample concentration that can capture 50% of the free radicals from DPPH. It was determined for several samples, such as: ascorbic acid, free Cur (UV irradiated and non-irradiated), and Cur-loaded in the micelles (UV irradiated and non-irradiated), from the graph I% vs. concentration.

### 2.7. In Vitro Hemolysis Assay

The hemolytic potential of the drug-free PMs was evaluated using a spectrophotometric method adapted from Rata et al. [17]. In these assays, blood from the healthy human volunteer was collected and treated with the PMs, after institutional ethical clearance and appropriate informed consent. In total, 5 mL anti-coagulated blood was centrifuged at 2000 rpm (RCF = 381× *g*) for 5 min and washed with normal saline solution several times to completely remove the plasma and obtain erythrocytes. Then, the purified erythrocytes were re-suspended in normal saline solution to obtain 25 mL of erythrocytes suspension. PMs saline solution with different concentrations (0.5 mL) was added to 0.5 mL of erythrocytes suspension (final concentrations were 200, 300, 400, and 500 mg PMs/mL erythrocytes suspension). Positive (100% lysis) and negative (0% lysis) control samples were prepared by adding equal volumes (0.5 mL) of Triton X-100 and a standard saline solution. The samples were incubated at 37 °C for 180 min. The samples were slightly shaken once every 30 min to re-suspend the erythrocytes and PMs. After the incubation time, the samples were centrifuged at 2000 rpm (RCF = 381× *g*) for 5 min and 100 µL of supernatant was incubated for 30 min at room temperature to allow hemoglobin oxidation. Oxyhemoglobin absorbance in supernatants was measured spectrophotometrically (Nanodrop One UV-Vis Spectrophotometer, Thermo Fischer Scientific, Waltham, MA, USA) at 540 nm. All samples were analyzed in triplicate. The hemolytic percentage was calculated using Equation (5):

(5) $$Haemolysis\;\left(\%\right)=\frac{\left(A_{s}-A_{NC}\right)}{\left(A_{PC}-A_{NC}\right)}\times 100$$

where, *A_s_* is the absorbance of the sample; *A_NC_* and *A_PC_* are the absorbance values of the negative and positive control, respectively.

### 2.8. Protein Adsorption Tests

The protein adsorption method was adapted after the work of Nandhakumar et al. [31]. These tests were carried out in phosphate buffer (PBS) at pH 7.4 (specific to the blood). The protein used as a model was bovine serum albumin (BSA). A stock solution of BSA in PBS with a concentration of 4 mg/mL was prepared and the concentration of micelles in this solution was 1 mg/mL. Briefly, the micelles were mixed with the BSA solution at different ratios of different concentrations, such as 95:5, 90:10, 80:20, 60:40, and 50:50 *v*/*v*. The test was performed in microcentrifuge tubes and the final volume of the mixture was 1000 µl. The prepared samples were incubated for 2 h at 37 °C in the dark and centrifuged at 14,000 rpm for 40 min. The supernatant was separated and the amount of protein was determined at a wavelength of 660 nm. The protein concentration in the supernatant was determined using the calibration curve obtained from known BSA concentrations. The amount of protein adsorbed by the micelles was calculated as the difference between the initial amount of protein in the solution and the amount of protein determined by the method of Lowry et al. [32].

The results were expressed in mg of adsorbed albumin/g micelles and by the adsorption efficiency (AE%) calculated with Equation (6).

(6) $$AE\left(\%\right)=\frac{Amount\;of\;adsorbed\;BSA}{Total\;amount\;of\;BSA\;in\;1ml\;solution\;}\times 100$$

### 2.9. Investigation of the In Vitro Cytotoxicity

*In vitro* cytotoxicity of the drug-free PMs was assessed on the human dermal fibroblasts adult cell line (HDFa). Briefly, HDFa cells were cultured in Dulbecco modified Eagle medium (DMEM), supplemented with 10% FBS, an antibiotic cocktail consisting of 1% (*v*/*v*) penicillin–streptomycin and 1% (*v*/*v*) non-essential amino acids. The medium was changed every day. Cell incubation was performed at 37 °C in a humidified atmosphere of 5% CO_2_ in air. Cells were allowed to grow in 2 culture flasks to 80% confluence and then were trypsinized with 0.25% trypsin solution at 37 °C for 3 min, followed by the addition of a fresh medium at room temperature to neutralize trypsin. The concentration of cells was 1 × 104 cells/cm^2^. After centrifugation and re-suspension in fresh medium, the viable cells were plated in flasks, and allowed to settle and attach for 24 h prior to the treatment. After that, the cells were treated with PMs, at different concentrations (200, 300, 400, and 500 μg/mL). Microscopic analysis was performed on the inverted optical microscope (CKX41, Olympus, Tokyo, Japan) with a built-in camera. Fibroblasts viability after 72 h of incubation in culture medium conditioned with the tested PMs was determined using the 0.4% trypan blue exclusion test and EVE™ Automated cell counter.

### 2.10. Statistical Analysis

Results are presented as the means ± SDs. The Student’s *t*-test was used for assessing unequal variance. A 2-tailed *p*-value less than 0.05 was considered significant.

## 3. Results

The micellization, as a function of pH, of this P2VP_90_-b-PEO_398_ block copolymer, was already studied in the absence of drugs. It was of interest to investigate the behavior of this micellar system, as a smart drug delivery system, as it was concluded that the micellization process is pH-dependent and starts at pH values higher than 4.5, which is the pKa of the P2VP sequence [24].

In the following, the influence of encapsulation and the release kinetics of two very different drugs, naturally-based Cur and synthetic 5-FU, and the hemocompatibility and the cytotoxicity of these PMs will be examined in order to evaluate their potential applicability as biocompatible drug nanocarriers.

### 3.1. Size of the Micellar Systems

**Figure 1:**
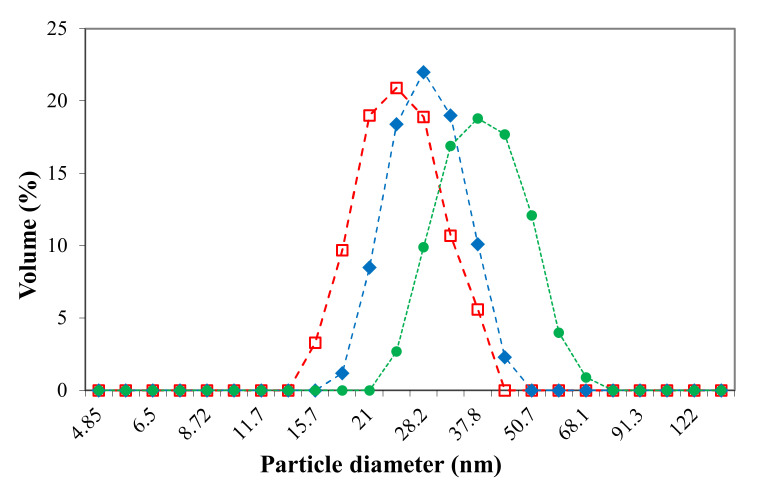
Volume size distributions for drug-free polymeric micelles (PMs; squares), Cur-loaded PMs (circles), and 5-FU-loaded PMs (diamonds) in PBS (pH = 7.4) at a concentration of 0.1 wt % and 37 °C.

In this paragraph, the micellar sizes are investigated, by DLS, as a function of the loaded drug, Cur or 5-FU. These free and drug-loaded micellar systems were obtained by the dialysis method, and the volume size distribution, determined by DLS in PBS at 37 °C and a pH value of 7.4, was monomodal as illustrated in Figure 1. The intensity averaged particle diameter (Z-average) and polydispersity index (PDI) values, respectively, are given in Table 1 for two temperature values, 25 and 37 °C, respectively.

In agreement with a previous study, it could be confirmed that this type of amphiphilic block copolymer forms, at pH values higher than 4.5, “frozen-in” micelles with a P2VP hydrophobic core and a PEO hydrophilic corona [24]. It can be noticed from Figure 1 that the loading process of the two drugs does not affect the size distribution of the micellar system. However, the drug loading leads to an increase in the micellar size, this increase being more pronounced in the case of Cur with respect to 5-FU. This behavior can be explained by the higher molar mass of the Cur with respect to 5-FU but also to the higher drug loading efficiency of Cur (see Table 2).

The values provided in Table 1 are correlated with the size distributions illustrated in Figure 1 and show that the loading of the Cur has an important effect on the micellar size, this effect being less pronounced for the loading of 5-FU. Moreover, it appears that the size of drug-free micellar systems at 25 °C in PBS (pH = 7.4) is in the same range as those previously obtained in distilled water at pH = 7 [24]. Furthermore, from Table 1, it also can be noticed that the PDI values of all studied micellar systems are smaller than 0.1, which is a direct proof of the fact that these systems have a very narrow polydispersity in size and a spherical shape. Finally, it can be observed from Table 1 that the temperature increase from 25 to 37 °C, which is the body temperature, leads to a decrease of the micellar size, since the compatibility of PEO sequences with water diminishes as temperature increases. From the DLS results it can be concluded that both Cur and 5-FU-loaded micellar systems can be used as injectable drug delivery systems as the micellar sizes are smaller than 100 nm. However, due to the blood rheological properties, these nanosized drug delivery systems are forced to stay on the vessel axis, i.e., far from the vessel wall, hindering the extravasation [33].

### 3.2. Fourier Transform Infrared Spectroscopy Analysis

Fourier transform infrared spectroscopy (FTIR) provides useful information about chemical bonding, molecular structure, and drug loading in multi-component systems. The IR spectrum for a physical mixture will be the sum of the spectra of the individual compounds, whereas for systems wherein some types of interactions (electrostatic, hydrogen bonds, etc.) occur, there will be changes in the IR spectra such as shifts in absorption bands, band broadening, and the appearance of new absorption bands. In this study FT-IR analysis was applied in order to examine the possible interactions between the copolymer and the two drugs (Cur and 5-FU) of the prepared micellar drug delivery systems.

Cur and 5-FU-loaded lyophilized PMs were analyzed by FTIR in comparison with free copolymer, Cur, and 5-FU in order to qualitatively evaluate the loading of these two drugs. The peaks assignments are provided in Tables S1 and S2 for Cur and 5-FU-based systems, respectively. From Table S1, it clearly appears that the shift of the characteristic peaks of Cur, corresponding to the aromatic C=C stretching, from 1628 to 1624 cm^−1^, is a direct proof of some interactions between Cur and copolymer, without any chemical composition change of the drug after loading. This result is in concordance with those founded by Gunathilake et al. for Cur loaded in chitosan hydrogels [34]. As also suggested by Davis et al. [35], the shifts of the C=O stretching, δ(CCC), and δ(CCC) in plane bending from 1510 to 1512 cm^−1^ can be an evidence for the successful incorporation of Cur into the PMs. Even if the majority of the characteristic peaks of 5-FU are overlapped with those of the copolymer, in the spectrum of 5-FU-loaded PMs, the shift of the C=O stretching peak from 1661 to 1568 cm^−1^ can be observed, as can the shifts of the ν_C-N_, ν_C-F,_ and ν_N-H_ stretching peaks from 948, 880, and 752 to 961, 842, and 748 cm^−1^, respectively. These shifts indicate that weak intermolecular interactions may occur during the loading of the 5-FU into the PMs.

### 3.3. Drug Encapsulation Efficiency

Having in mind that two very chemically different drugs were used in this study, it was of interest to determine both their drug encapsulation efficiency (DEE) and drug loading efficiency (DLE). An identical procedure was used for both drugs and the obtained results are presented in Table 2.

As shown in Table 2, both Cur and 5-FU can be incorporated into micelles with relatively high encapsulation and loading efficiencies. Moreover, it clearly appears that Cur was encapsulated more efficiently than 5-FU, and this feature is due to its more pronounced hydrophobic character, which gives rise to stronger hydrophobic interactions with the P2VP micellar core.

### 3.4. In Vitro Drug Release Kinetics

**Figure 2:**
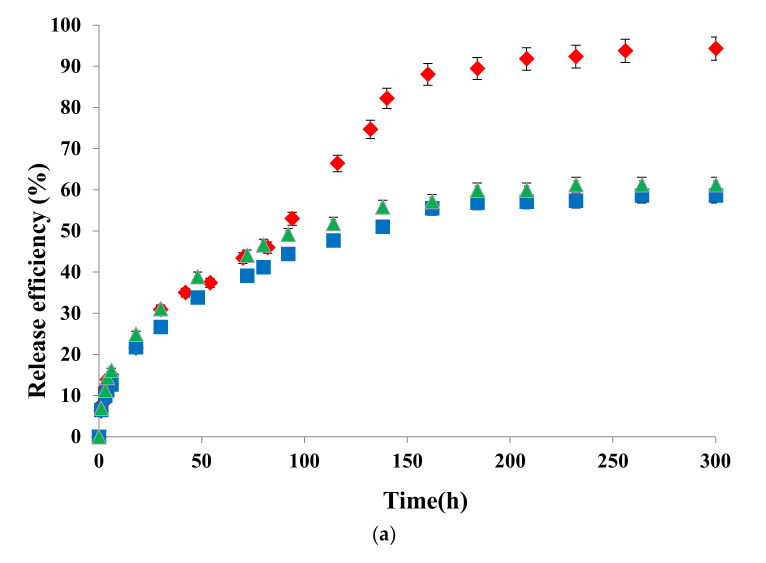

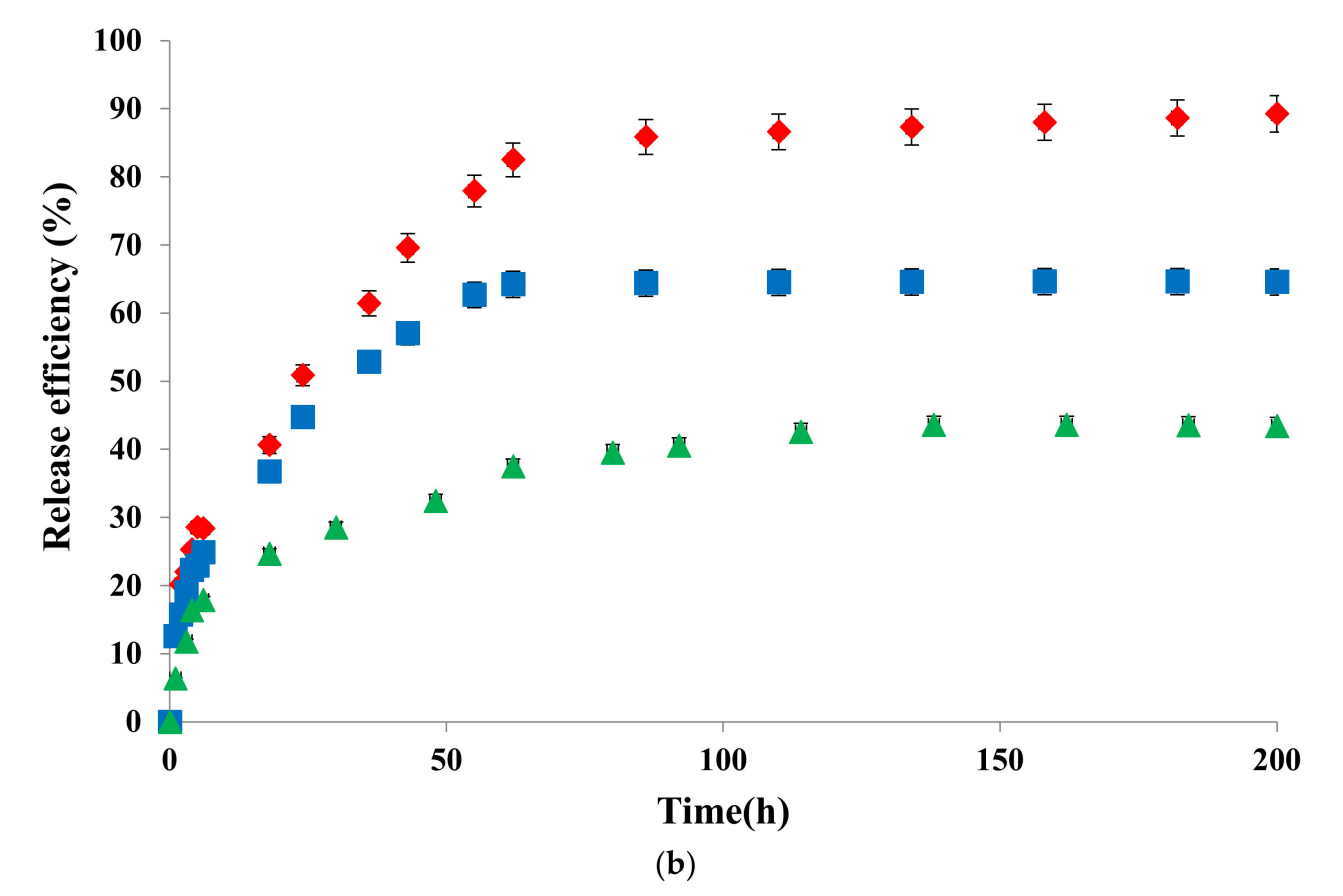
Curcumin (**a**) and 5-FU (**b**) release efficiency as a function of time at pH 2 (diamonds), pH 6.8 (squares), and pH 7.4 (triangles) and 37 °C.

The release kinetics of 5-FU and Cur from the PMs were evaluated in three different pH environments: at pH = 2 (simulates the pH of the gastric environment), pH = 6.8 (simulates the pH of the intestinal environment), and pH = 7.4 (simulates the pH of the blood). The samples were kept in the dark on a water bath, at a temperature of 37 °C, the release kinetics of the loaded drugs (expressed in terms of release efficiency, %) being studied until equilibrium. The method of analysis used in this study (dynamic dialysis) is the most appropriate in the kinetic examination of the release processes from nanocarriers, especially from micelles, which are characterized by a very small size. Although the dialysis membrane reduces the driving force of drug’s transport (based on the concentration gradient) and as a result can cause a seemingly slower release rate, the method is widely used as it allows a sufficiently accurate evaluation of the process and especially a correct comparison of different polymer/drug systems [36]. Figure 2 illustrates the release efficiencies for both drugs as a function of time for all three pH values.

The data illustrated in Figure 2 show that for both drugs the release process is influenced by the pH. The highest release efficiency is achieved at a strong acidic pH (pH = 2). At this pH value, the P2VP sequences from the “frozen-in” micellar core are protonated, leading thus to the formation of unimers and disintegration of the PMs [24]. This phenomenon has, as a consequence, the rapid releasing of the drugs. In the case of Cur-loaded micelles, it seems that the duration of the protonation process is influenced by the hydrophobic–hydrophobic interactions between Cur and P2VP sequences, which leads to a delay in the disintegration process of the micelles. This behavior is less pronounced in the case of 5-FU. Moreover, the release efficiencies of Cur from PMs are almost identical at pH 6.8 and 7.4. At these pH values, the solubility of Cur is increased, as Cur turns in the phenolate form (sodium salt), leading thus to the intensification of the diffusion process through the micellar core. On the contrary, a clear effect of the pH increase on the release efficiency is observed in the case of 5-FU-loaded PMs. This effect was also observed by Li et al. [37] for the release of 5-FU from nanoparticles based on poly(ethylene glycol)-b-poly(benzyl-L-glutamate) (PEG-b-PBLG). At this point it is also important to notice that 5-FU can form hydrogen bonds with the nitrogen of the P2VP groups [38,39]. Although it is not very pronounced, for all the analyzed samples, a less intense “burst effect” was observed in Figure 2. If in the case of nanospheres this effect is caused by the rapid release of the drug adsorbed on the surface of the particle or in its surface layers, in the present case it may be due to the rapid release of the drug “tangled” in the hydrophilic PEO micellar corona, possibly by hydrogen bonds.

Finally, from Figure 2, it also appears that Cur release is slower with respect to 5-FU, a logical effect due to the lower solubility of Cur in aqueous media. In addition, this difference can be explained by the stronger hydrophobic character of the Cur, which determines stronger hydrophobic–hydrophobic interactions with the P2VP micellar core. As a result, the equilibrium of the release process is reached at longer durations of around 300 h, compared to 200 h for 5-FU. The values of the constant of the release process, k, lower in the case of Cur (see Table 3), support the above conclusion.

Information on the mechanism of transport and release of Cur and 5-FU from PMs was obtained using the Ritger–Peppas kinetic model [29]. The kinetic curves for Cur-loaded and 5-FU-loaded PMs are provided in Figure S1 (Supplementary Materials). A maximum release efficiency of 60% was considered for plotting these curves, as recommended by the Ritger–Peppas theory [29].

The n values, given in Table 3, indicate that a Fickian diffusion mechanism describes the release of drugs from these spherical nanomicelles. In fact, these PMs are essentially of nanogel type, as they have, at these experimental conditions, a “frozen in” P2VP micellar core. The release rate constant (k) values depend on the physical and structural properties of both drug and the polymeric matrix [40]. The k values from Table 3 indicate that the rate of Cur release from PMs is lower than that of 5-FU, which is in concordance with the graphical illustration from Figure 2. Moreover, the higher values of R^2^ are a clear proof that these results fit perfectly the Ritger–Peppas kinetic model.

From these tests it can be concluded that the rate of drug release from PMs is controlled by the pH and depends on drug structure, solubility, molar mass, and the interactions that may occur with the hydrophobic P2VP micellar core.

### 3.5. Antioxidant Activity of Curcumin

The examination of the antioxidant activity of Cur was necessary, since the administration of these drug delivery systems, by intravenous injection, involves its prior sterilization. A practical method to carry out sterilization is by UV irradiation, which is a process that can cause degradation not only of the PMs, but especially of the loaded drugs. UV exposure time seems to be one of the most important factors affecting the properties of the material after sterilization. For example, Fischbach et al. [41] reported that an exposure duration of 2h to UV radiation was sufficient to effectively sterilize polymer films based on poly(D,L-lactic acid)-poly(ethylene glycol)-monomethyl ether di-block without causing significant changes to the copolymer, while exposure over a longer period of 5 to 24 h can cause profound changes in the properties of the material, with considerable degradation of the PEG chains. Degradation of polyphenols reduces the effectiveness of their therapeutic effects and affects the use of these antioxidants in food/nutraceutical or pharmaceutical applications [42]. In most research studies, UV radiation is most commonly used for sterilization. Still, studies of Cur degradation have shown that approximately 50% of Cur has been degraded after 8 h of exposure [43].

**Figure 3:**
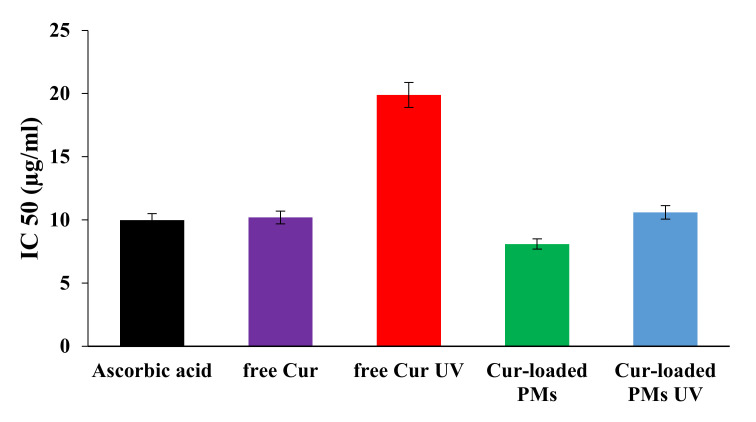
IC50 values for ascorbic acid, and non-irradiated and UV-irradiated free Cur and Cur-loaded PMs.

Figure 3 shows the IC50 values obtained after evaluating the antioxidant activity using the DPPH test for ascorbic acid, free Cur, and Cur-loaded in PMs (non-irradiated and irradiated samples with UV light were used for 30 min, λ = 365 nm). In principle, a low value of IC50 indicates a strong antioxidant character of Cur.

From Figure 3 it can be observed that the IC50 values for ascorbic acid, free Cur, and Cur-loaded in PMs from irradiated or non-irradiated UV micelles are very similar, which means that by encapsulation in the PMs, the Cur retains its antioxidant properties and the micelles have a protective role against Cur photo-degradation. For free Cur exposed to UV light, it was found that the IC50 value is clearly higher than that corresponding to non-irradiated free Cur, which obviously indicates its photo-chemical degradation in a proportion of over 80%. The results are consistent with those obtained by Chen et al. [44] which have found that the degradation of Cur loaded in micelles, based on hydroxyethyl starch, after 3 h, is maximum 20%, while for free Cur exposed to UV irradiation, the degradation occurs to the extent of around 80%. At this point, it should be mentioned that the by-products of photo-chemical degradation of Cur (ferulic acid, ferulic aldehyde, vanillin, etc.) can have an antioxidant activity due to the presence of the phenolic -OH group in their structure [43].

It can be concluded that the loading of the Cur into the PMs based on P2VP_90_-b-PEO_394_ block copolymer has as a consequence its protection against photo-chemical degradation for a duration of exposure compared to that required for sterilization.

### 3.6. In Vitro Hemolysis Assay

**Figure 4:**
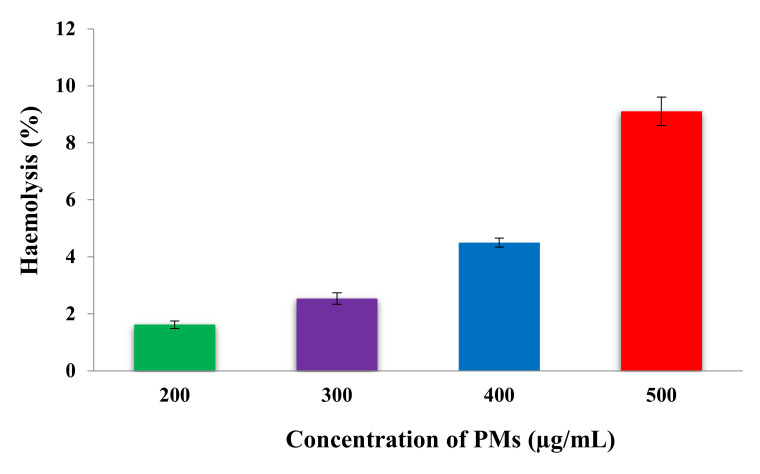
In vitro hemolysis evolution as a function of the concentration of PMs.

An important feature in the development of injectable drug delivery systems is to determine their ability to cause hemolysis. Hemolysis is defined as being the disintegration of red blood cells with the release of hemoglobin and other internal components into the surrounding fluid. If this disintegration occurs to a significant degree of red blood cells in the body, it can lead to dangerous pathological conditions. Therefore, the micellar system based on the pH-sensitive P2VP_90_-b-PEO_394_ block copolymer was evaluated, at different concentrations from 200 to 500 μg/mL, in order to study the occurrence of lysis in human erythrocytes. The results of the hemolysis assay, obtained after 180 min, as a function of PMs concentration, are shown in Figure 4, and were expressed as means ± SDs (n = 3).

The obtained results from Figure 4 show that the hemolytic percentage increases with increasing concentration of PMs. From the literature data it appears that a system is hemocompatible if the hemolysis percentage is under 5% [45]. Therefore, the information obtained from Figure 4 suggests that these micellar systems, based on pH-sensitive P2VP_90_-b-PEO_394_ block copolymer, have good compatibility with the bloodstream until a concentration of 400 μg/mL. At this point, it is essential to note that this concentration value is much higher than the normal in vivo concentration which might be reached after an intravenous administration. From this test, it can be concluded that the obtained pH-sensitive micellar system can be used as an injectable drug delivery system.

### 3.7. Protein Adsorption Tests

The in vivo stability and blood circulation time of polymeric micellar structures are also affected by the adsorption of proteins present in plasma. Biological proteins can bind to the surfaces of different types of particles through hydrophobic, electrostatic, or chemical interactions. The layer thus formed induces new physicochemical characteristics to the particles and influences both their properties and the encapsulated drug (membrane transport, half-life in the systemic circulation, and their targeting) [46]. Moreover, adsorption of proteins on PMs surfaces can enhance the uptake of these carriers by the reticuloendothelial system (RES) and lead to their early removal from blood circulation [47,48].

**Figure 5:**
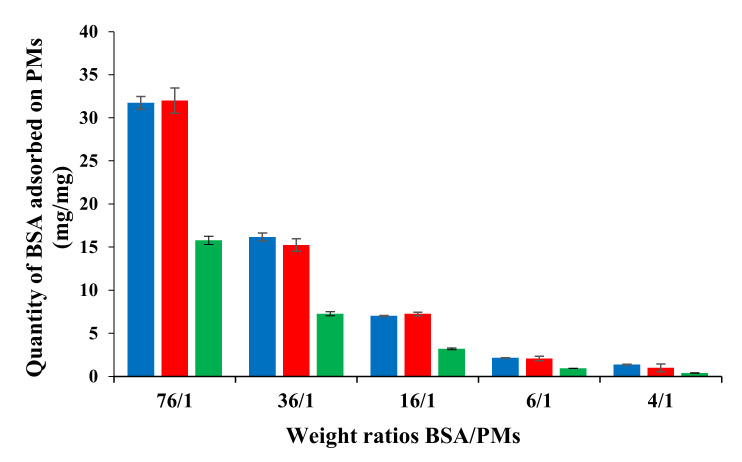
Amount of BSA adsorbed on free PMs (blue), Cur-loaded PMs (red), and 5-FU-loaded PMs (green) as a function of the weight ratio BAS/PMs.

Bovine serum albumin (BSA) was chosen as a model protein for this study because it is structurally similar to human serum albumin (HSA), which has the highest plasma concentration. The test carried out in order to analyze the adsorption of proteins on the surfaces of the PMs was performed for free and Cur-loaded PMs, using five weight ratios between albumin and PMs (Table 4). The amount of BSA adsorbed on the PMs (Figure 5) was determined after two hours, during which time the albumin solution with micelles was maintained at 37 °C in an oven in closed containers.

From Table 4, it appears that, at high weight ratios BSA/PMs, the absorption efficiency (AE%) of BSA for both free and Cur-loaded PMs showed similar values ranging between 42% and 45%. The Student t-test shows that the differences between the results obtained for these samples at these weight ratios have no statistical significance *p* > 0.05. Moreover, for low weight ratios, 6:1 and 4:1, the values of AE% are also similar and vary between 34% and 36%. Additionally, in this case, the differences between the values of AE% have no statistical significance.

For Figure 5, it can be noted that the amount of BSA adsorbed decreases, as expected, with the decrease of the weight ratio between BSA and PMs. Moreover, the amount of BSA (in mg/mg) absorbed on the 5-FU-loaded PMs is systematically smaller than that absorbed on the free and Cur-loaded PMs. However, by taking into account the molar masses of Cur (368.38 g/mol) and 5-FU (130.07 g/mol), respectively, and also the loading efficiency, we can calculate the numbers of moles of both 5-FU (0.447 moles) and Cur (0.174 moles) in the hydrophobic micellar core. From this calculation, it can be supposed that the higher number of loaded 5-FU moles has a protective effect against the adsorption of BSA by reducing the hydrophobic sites available for this adsorption. The differences between the obtained results have a statistical significance *p* < 0.05 all the samples. A similar trend was observed by Nandhakumar et al. [31], for the adsorption of protein on the poly(ε-caprolactone) nanoparticles. Another observation is related to the fact that the adsorption of the BSA is not influenced by the encapsulation of Cur within the PMs, similar values being obtained for both free and Cur-loaded PMs.

Biological proteins can be adsorbed on the surfaces of nanoparticles through hydrophobic, electrostatic, or chemical interactions leading to the formation of a protein corona on their surface [46].

Even if the BSA has an amphoteric character, the protein adsorption in this case is not induced by the electrostatic interactions. The isoelectric point of the protein being at pH = 4.5, the amino groups are not protonated in the experimental conditions used for this test (0.1M phosphate buffer at pH = 7.4). Therefore, it can be admitted that there are no electrostatic interactions between the BSA and PMs. In addition, the PEO sequences of the micellar corona of these PMs have a non-ionic character, zeta potential value at pH = 7 is equal to 1.2 mV, and thus it can be supposed that the steric repulsion will limit the protein adsorption onto the micellar surface.

The protein adsorption observed for the micellar system investigated in this study might be due to the fact that the length of the PEO chains from the micellar corona is not sufficient in order to entirely block the hydrophobic adsorption sites for the protein on the micellar core. However, these results are similar to those obtained by Garg et al. [49] for the adsorption of fetal bovine serum on PCL-b-PEO and PBCL-b-PEO block copolymer micelles, and therefore it can be concluded that the amount of protein adsorbed does not alter the application of these nanomicelles as injectable drug delivery systems.

### 3.8. Investigation of the In Vitro Cytotoxicity

**Figure 6:**
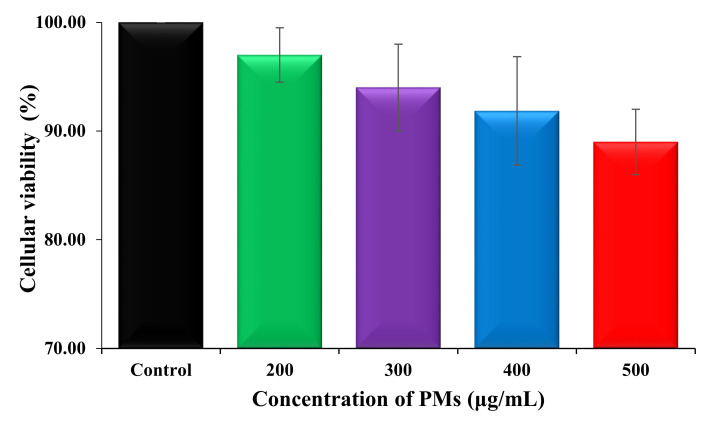
Evolution of the HDFa cellular viability after a treatment of 72 h with different concentrations of drug-free PMs.

**Figure 7:**
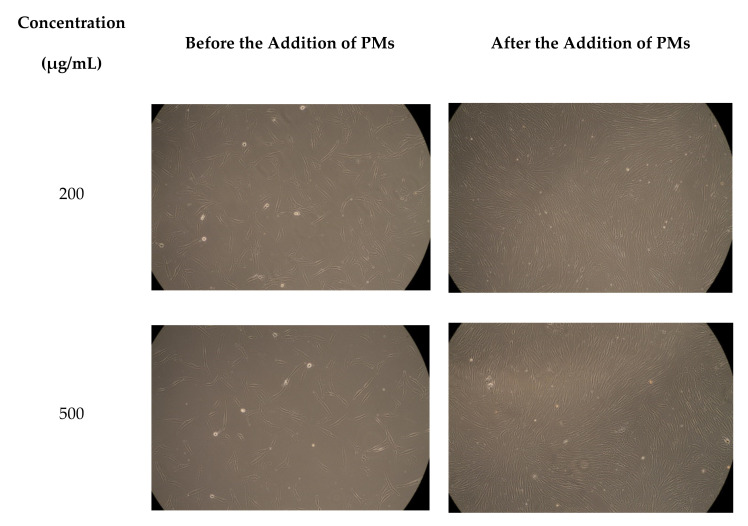
Micrographs of the fibroblast cells before and after the treatment with the PMs.

Cytotoxicity is one of the most important indicators for the biological evaluation of in vitro studies of different materials and the determination of the cell viability is the most common analysis in the evaluation of cytotoxicity of biomaterials. In this study, the cellular viability of blank micelles was investigated, after 72 h, at four concentrations between 200 and 500 μg/mL, and the obtained results are given in Figure 6. Figure 7 shows the micrographs which were taken for the fibroblast cells before and after the addition of PMs.

As can be seen in Figure 6, fibroblast cells treated with blank PMs, at different concentrations, such as: 200, 300, 400, and 500 μg/mL, exhibit good cellular viability ranging from 90% to 97%. These results clearly demonstrate that the drug-free micellar systems, based on pH-sensitive P2VP_90_-b-PEO_394_ block copolymer, do not have cytotoxic effects even at a high concentration and can be safely used as drug delivery systems.

## 4. Conclusions

Two drugs, Cur and 5-FU, were loaded into the micellar P2VP core of a pH-sensitive P2VP_90_-b-PEO_398_ block copolymer by a dialysis method. By DLS, it was demonstrated that the micellar sizes of the drug-loaded systems are higher than that of the drug-free PMs and that this increase is correlated with the molar mass of the loaded drugs. In fact, Cur, having the highest molar mass, has the most important effect on the increase of the micellar size. This higher increase of the micellar size for the Cur-loaded micelles can also be explained by the higher drug loading efficiency of Cur with respect to 5-FU. Moreover, the very narrow PDI values indicate that these micelles are spherical.

The release efficiency of both drugs was highly influenced by the pH value of the release medium; the highest release efficiency, more than 90%, being observed at pH 2 where the P2VP micellar core, was protonated, leading thus to the destruction of the PMs. In addition, it appears from the k values, calculated using the Ritger–Peppas equation, that 5-FU is released at a higher rate than Cur, at all pH values. This behavior can also be explained by the smaller molar mass of the synthetic drug compared with that of Cur.

Furthermore, it was clearly demonstrated by the determination of the IC50 values that these smart PMs have the ability to protect the Cur from the photo-chemical degradation under UV irradiation. This feature is important from a practical point of view as it allows the sterilization of the drug-loaded micellar system by UV without decreasing the anti-oxidant activity of the loaded Cur.

Another important conclusion is related to the fact that these smart PMs are hemocompatible and biocompatible, at concentrations high as 400 μg/mL.

Finally, due to the presence of the non-ionic PEO corona, the adsorption of BSA protein on the surface of the micelles is limited, and this effect might allow a higher in vivo blood circulation time.

All these results suggest that this micellar system based on pH-sensitive P2VP_90_-b-PEO_394_ block copolymer can be safely used as smart drug delivery systems with practical application in the biomedical field.

As a perspective, it will be of interest to study the dual encapsulation and release of Cur and 5-FU from this type of pH-sensitive micellar system in order to evaluate the synergetic effect of these two drugs.

## Figures and Tables

**Table 1:** Z-average and polydispersity index (PDI) values of free and drug-loaded micellar systems based on the pH-sensitive P2VP_90_-b-PEO_394_ block copolymer at a concentration of 0.1 wt% in PBS (pH = 7.4) and two temperature values, 25 and 37°C, respectively.

T (°C)	Drug-Free PMs		Cur-Loaded PMs		5-FU-Loaded PMs	
	Z-Average (nm)	PDI	Z-Average (nm)	PDI	Z-Average (nm)	PDI
**25**	63.0 ± 0.5	0.05	104.5 ± 0.3	0.07	64.5 ± 0.4	0.08
**37**	59.8 ± 0.4	0.04	84.7 ± 0.6	0.06	63.8 ± 0.5	0.08

**Table 2:** Drug encapsulation efficiency (DEE) and drug loading efficiency (DLE).

Loaded Drug	DEE (%)	DLE (%)
**Cur**	70.4	6.4
**5-FU**	64.0	5.8

**Table 3:** Basic kinetic parameters of the process of Cur and 5-FU kinetics released from PMs using the Ritger–Peppas kinetic model.

pH Value	Drug	n	k × 10^−2^	R^2^
**2**	**Cur**	0.45	6.62	0.990
	**5-FU**	0.40	14.23	0.986
**6.8**	**Cur**	0.43	6.27	0.999
	**5-FU**	0.40	12.25	0.996
**7.4**	**Cur**	0.41	7.51	0.995
	**5-FU**	0.37	8.01	0.965

**Table 4:** Experimental data and adsorption efficiency values of the BSA on the PMs.

Experimental ^a^		BSA Solution Volume (µL)	PMs Solution Volume (µL)	Weight Ratio BSA/PMs (µg/µg)	Adsorption Efficiency (%)
**Cur free PMs**	1	950	50	76/1	42 ± 0.97
	2	900	100	36/1	45 ± 1.28
	3	800	200	16/1	44 ± 0.37
	4	600	400	6/1	36 ± 0.43
	5	500	500	4/1	34 ± 1.18
**Cur-loaded PMs**	C1	950	50	76/1	42 ± 1.94
	C2	900	100	36/1	42 ± 2.04
	C3	800	200	16/1	45 ± 1.28
	C4	600	400	6/1	35 ± 4.2
	C5	500	500	4/1	34 ± 2.7

^a^ The total volume of the analyzed solution (consisting of PMs and protein) was equal to 1 mL.

## Supplementary Materials

The following are available online at https://www.mdpi.com/2073-4360/12/7/1450/s1.
