# Supplementary material for: Drug Delivery System Based on pH-Sensitive Biocompatible Poly(2-vinyl pyridine)-b-poly(ethylene oxide) Nanomicelles Loaded with Curcumin and 5-Fluorouracil
Source: Polymers (Basel). 2020 Jun 28;12(7):1450. doi: 10.3390/polym12071450 (PMC7408444; doi:10.3390/polym12071450)
Supplement: Supplementary file 1 [file polymers-12-01450-s001.pdf]

Supplementary Materials

Drug Delivery System Based on pH-Sensitive Biocompatible Poly(2-vinyl pyridine)-b-poly(ethylene oxide) Nanomicelles Loaded with Curcumin and 5-Fluorouracil

Camelia-Elena Iurciuc-Tincu [1,2], Monica Stamate Cretan [1], Violeta Purcar [3], Marcel Popa [2,4], Oana Maria Daraba [5], Leonard Ionut Atanase [5,*] and Lacramioara Ochiuz [1]

[1] Faculty of Pharmacy, "Grigore T. Popa" University of Medicine and Pharmacy, Department of Pharmaceutical Technology, University street, no. 16, 700115 Iaşi, Romania; camelia_tincu83@yahoo.com (C.-E.I.-T.); cretanmonica@yahoo.com (M.S.C.); ochiuzd@yahoo.com (L.O.)

[2] "Gheorghe Asachi" Technical University, Faculty of Chemical Engineering and Protection of the Environment, Department of Natural and Synthetic Polymers, 700050 Iaşi, Romania; marpopa2001@yahoo.fr

[3] National R&D Institute for Chemistry and Petrochemistry—ICECHIM, Splaiul Independentei 202, 6th district, Bucharest, Romania; purcarvioleta@gmail.com

[4] Academy of Romanian Scientists, Splaiul Independentei Street No. 54, 050085 Bucharest, Romania

[5] Faculty of Dental Medicine, "Apollonia" University of Iasi, Pacurari street, no. 11, 700355 Iași, Romania; maria.mary2019@yahoo.com

* Correspondance: leonard.atanase@yahoo.com or leonard.atanase@univapollonia.ro

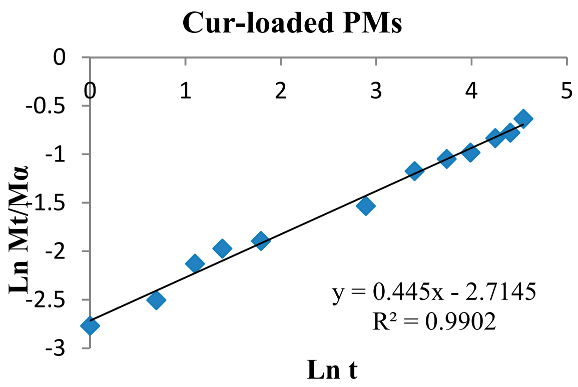

(a)

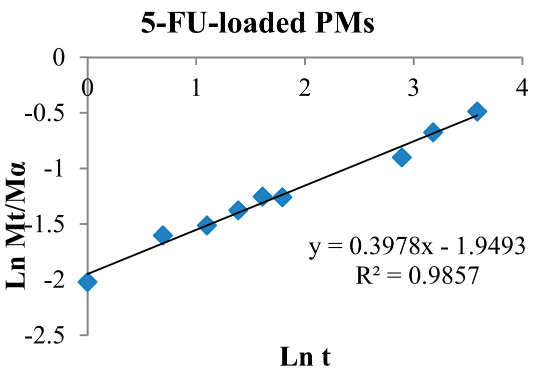

(a')

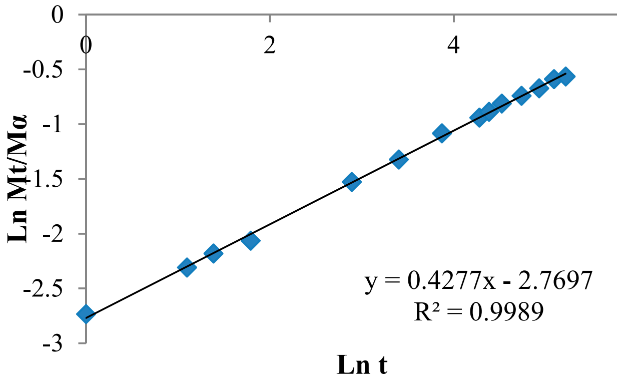

(b)

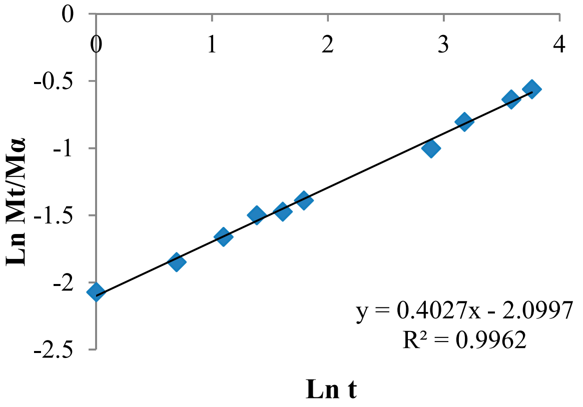

(b')

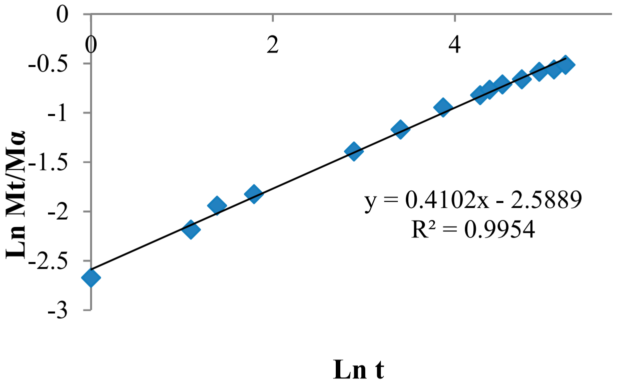

(c)

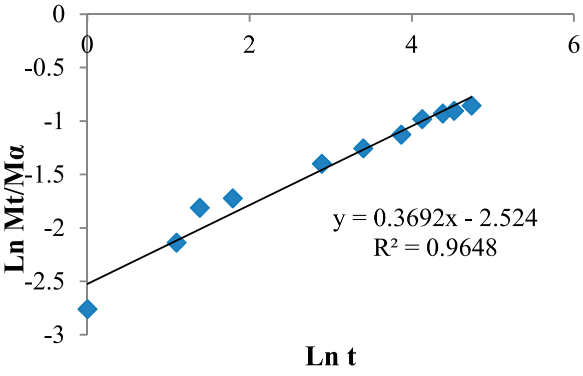

(c')

Figure S1: Graphical representations of ln(release efficiency) vs ln(time) for Cur-loaded PMs and 5-FU-loaded PMs at pH 2 (a, a'); 6.8 (b, b') and 7 (c, c')

Table S1: Peaks assignments for free Cur, free P2VP$_{90}$-*b*-PEO$_{398}$ copolymer, and Cur-loaded PMs obtained by FTIR

**Groups**	**Frequencies (cm^{-1})**		
	**Curcumin**	**P2VP$_{90}$-*b*-PEO$_{398}$ copolymer**	**Cur-loaded PMs**
ν_{OH} stretching	3511	3100-3600	3100-3600
ν_{CH}	3000-2930	2882	2882
ν_{CH2}	3000-2930	2882	2882
$\nu_{C=C}$ stretching	*1628*	-	*1624*
$\nu_{C=C}$ stretching	1600	1590	1590

$\nu_{C=O}$ stretching	1600	1568	1568
$\nu_{C=O}$ stretching	*1510*	-	*1512*
δ_{CH2}	-	1469	1467
ν_{CH} stretching	-	1434	1434
δ_{C-N}	-	1360	1360
ν_{CH3} stretching	-	1342	1342
ν_{C-O} (in phenol) stretching	1280	1280<br>1241	1280<br>1241
ν_{C-O-C} stretching	-	1147	1146
δ_{C-N}	-	1103	1101
ν_{CH2}	-	1061	1061
δ_{C-H}	*975*	-	*994*
δ_{C-N}	-	961<br>842	961<br>842
ν_{N-H} stretching	788	748	748
δ_{C-CH}	788	748	748

Table S2: Peaks assignments for free 5-FU, free P2VP$_{90}$-*b*-PEO$_{398}$ copolymer, and 5-FU-loaded PMs obtained by FTIR

**Groups**	**Frequencies (cm^{-1})**		
	**5-FU**	**P2VP$_{90}$-*b*-PEO$_{398}$ copolymer**	**5-FU-loaded PMs**
ν_{OH} stretching	-	3100-3600	3100-3600
ν_{CH} stretching	2800-3100	2882	2882
δ_{N-H}	2800-3100	2882	2882
ν_{CH2} stretching	2800-3100	2882	2882
$\nu_{C=C}$ ring stretching vibrations	1661	1590	1590
$\nu_{C=O}$ stretching	1661	1568	1568
δ_{CH2}	1450	1469	1469
ν_{CH}	1430	1434	1434
δ_{C-N}	1349	1360	1360
ν_{CH3} stretching	1349	1342	1342
ν_{CH}	1246	1280	1280
ν_{CH2}	1246	1241	1241
ν_{C-O-C} stretching	1182	1147	1147
δ_{C-N}	1182	1103	1101
ν_{CH2}	-	1061	1061
ν_{C-N} stretching	948	961	961
ν_{C-F} stretching	880	842	842
ν_{N-H} stretching	752	748	748
ν_{C-CH} stretching	752	748	748
